# SOD1 D91A variant in the southernmost tip of Europe: a heterozygous ALS patient resident on the island of Gozo

**DOI:** 10.1038/s41431-021-00975-x

**Published:** 2021-10-07

**Authors:** Maia Farrugia Wismayer, Andrew Farrugia Wismayer, Adrian Pace, Neville Vassallo, Ruben J. Cauchi

**Affiliations:** 1 Centre for Molecular Medicine and Biobanking, Biomedical Sciences Building, University of Malta, Msida, Malta; 2 Department of Physiology and Biochemistry, Faculty of Medicine and Surgery, University of Malta, Msida, Malta; 3 Department of Neurology, Gozo General Hospital, Victoria, Gozo Malta

**Keywords:** Motor neuron disease, Neurodegeneration

## Abstract

Amyotrophic lateral sclerosis (ALS) is frequently caused by mutations in the *SOD1* gene. Here, we report the first *SOD1* variant in Malta, an archipelago of three inhabited islands in southern Europe. We describe a patient with a sporadic form of ALS living on the island of Gozo in which the heterozygous *SOD1* c.272A>C; p.(Asp91Ala) variant was detected. The patient had a late onset (79 years), sensory impairments and rapid disease progression culminating in respiratory failure. ALS has not yet developed in any of the three additional family members in which the D91A variant was identified. None of the healthy controls from the Maltese population were found to carry this variant. This report underscores the high prevalence of the D91A variant in Europe, despite the presence of a North-South gradient in its frequency, and confirms that this variant can be associated with dominant cases in Mediterranean countries.

## Introduction

Amyotrophic lateral sclerosis (ALS) is an adult-onset, rapidly progressing, neurodegenerative disease. Onset is typically accompanied by clinical signs of upper and/or lower motor neuron degeneration with patients showing weakness in the bulbar muscles, only the limbs, or both regions simultaneously. Death usually occurs 2–3 years after clinical onset, mostly because of respiratory failure. ALS is classified as familial (fALS) in the presence of a clear family history of the disease and sporadic (sALS) when this is absent [1]. To date, variants in any of more than 40 genes have been reported to cause monogenic fALS with more than half of the cases explained by highly penetrant causal variants residing in *C9orf72* (23%), *SOD1* (19%), *TARDBP* (3%) or *FUS* (3%) genes [2].

*SOD1*, the first gene linked to ALS, encodes for the copper/zinc superoxide dismutase, a metalloenzyme that catalyses the dismutation of the superoxide radical to hydrogen peroxide and oxygen [3]. All *SOD1* deleterious variants show autosomal dominant inheritance except the p.Asp91Ala or D91A variant (also known as D90A; dbSNP^155^ ID rs80265967), which shows recessive inheritance, initially described in ALS cases from Sweden, Norway and Finland [4]. Nonetheless, heterozygous *SOD1* D91A ALS patients have now been described in various European populations [5] including those in southern Italy [6, 7]. *SOD1* D91A appears to have been a founder allele in Finno-Scandinavian populations [8, 9], hence explaining the high allele frequency in Finland and northern Sweden [10], and the spread of the allele in Europe could be the result of Viking conquests. *SOD1* D91A ALS patients have a heterogenous clinical phenotype with homozygotes displaying slow progression with long survival, while heterozygotes have a more variable clinical pattern [4]. Establishing whether *SOD1* D91A is pathogenic in the heterozygous state in particular populations, is imperative, considering that patients can be better informed about whether they can benefit from gene-specific treatments presently in development including *SOD1*-specific antisense oligonucleotide, Tofersen [11].

Gozo (known by inhabitants as ‘Għawdex’) is the second-largest island of the Maltese archipelago (total area 67 km^2^), located at the southernmost tip of Europe. The population of Gozo presently numbers around 34,430, which is 6.7% of the total population of Malta (514,564). The Gozitan population was seeded around 5000 BC by settlers coming from neighbouring Sicily. More than 900 years ago, both Malta (including Gozo) and Sicily were ruled by the Normans, who descended from Norse Vikings [12]. The capital city of Gozo is Victoria (known by inhabitants as ‘*Rabat’*) and its population is the least homogenous compared to the other villages and towns of the island [13]. We have recently showed that Maltese ALS patients do not have deleterious variants in *C9orf72*, *SOD1*, *TARDBP* or *FUS* genes indicating that the most commonly mutated ALS genes globally do not have a major impact on the ALS population in Malta [14]. Here, we report the first *SOD1* variant in the Maltese ALS population. We describe a sALS patient living in Gozo in which the heterozygous D91A variant was detected.

## Materials and methods

### Study participants

On obtaining the index patient’s family history, other family members were interviewed and neurologically evaluated. For reference purposes, 27 (13 men, 14 women) healthy elderly subjects (mean age 70.6 ± 11.5 SD) were recruited. Written informed consent was obtained from all participants.

### Genome sequencing and mining

DNA extraction and whole-genome sequencing was done as described previously [14]. The index patient’s genome was mined for variants in established ALS causative genes, restricting analyses to those with European minor allele frequency ≤0.01. Allele verification was performed by Sanger sequencing.

## Results

### Clinical findings

**Fig. 1:**
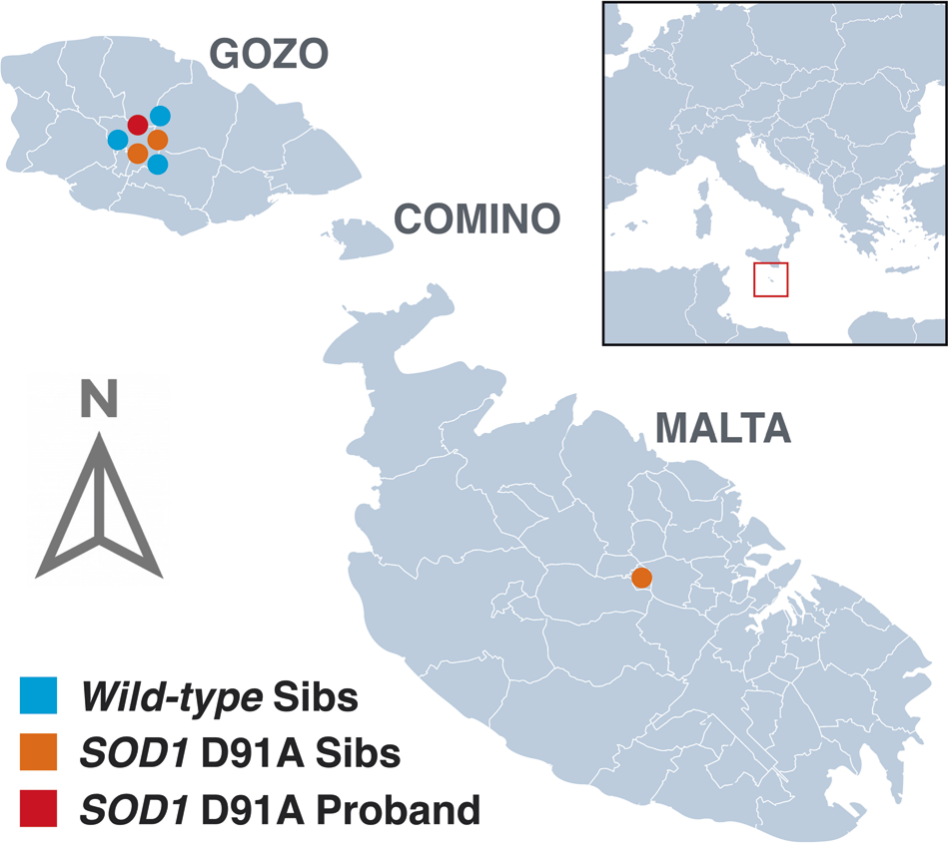
Geographical distribution of proband and siblings on the Maltese archipelago. *SOD1 D91A* proband resided in Victoria, the capital city of Gozo. Most sibs also reside in this locality. One sib has since relocated to mainland Malta.

**Fig. 2:**
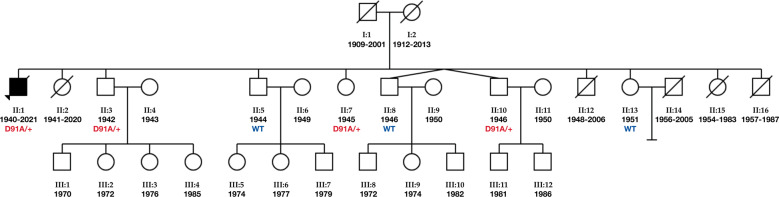
Three-generation family pedigree. The arrow indicates the proband. Circles are females whilst squares are males. A diagonal arrow indicates that the individual is deceased. Filled symbols indicate individuals affected with ALS. *SOD1* D91A allele status is indicated for the individuals analysed. In addition to the proband, *SOD1* D91A was identified in two brothers and a sister. The wild-type sequence was present in two brothers and a sister. Date of birth and date of death are indicated where available. Two brothers and two sisters of the index patient died at different ages from cancer. No biological material of the deceased individuals was available for analyses.

A 79-year-old male (II:1) living in Victoria (Fig. 1), was admitted to our department because of a several month history of right leg weakness, dragging right foot and deteriorating gait. Family history was negative for neurological diseases except for the proband’s mother (I:2) who died at 101 years from stroke (Fig. 2). The father (I:1) died at 92 years of age from natural causes. Neurological examination of the cranial nerves showed normal facial motor function, a hoarse dysphonic voice, no tongue fasciculations but difficulty with protruding the tongue out of the mouth. Limb examination revealed lower limb areflexia, flexor plantar reflex, lack of clonus, leg muscle atrophy, and monoparesis of the right lower limb with a partial foot drop. There were florid fasciculations over both biceps and triceps, bilateral anterior chest and back muscles without evident wasting. Sensory testing revealed reduced perception of light touch, pin prick and joint position at both feet. Cranial and spinal MRI detected no abnormalities. Electromyography and nerve conduction studies revealed fasciculations and denervation in 3 limbs, trunk and tongue. The patient had no swallowing issues and speech was mildly affected. ALS Functional Rating Scale-revised (ALSFRS-R) score was 36. He reported to have worked as a nurse for about 40 years prior to retiring. This occupation falls outside the risk category flagged by us in a recent study [15].

Progressive paraparesis ensued over the subsequent 12 months. Fasciculations gradually resolved followed by the emergence of mild upper motor neuron signs, increasing dysphonia but adequate swallowing. Upper limbs were relatively spared. The patient was admitted to hospital because of diminished appetite, weight loss, low mood and weakening voice. Mild dysphagia was identified and overnight oximetry showed nocturnal desaturation. Arterial blood gases were indicative of type 2 respiratory failure despite the absence of shortness of breath at rest. Some early accessory muscle use was noted. The patient was started on non-invasive ventilation. Unexpectedly, respiratory function deteriorated rapidly, partly due to non-compliance. Death occurred during the night from sudden cardiorespiratory arrest, one year from symptom onset.

### Genetic analysis

**Fig. 3:**
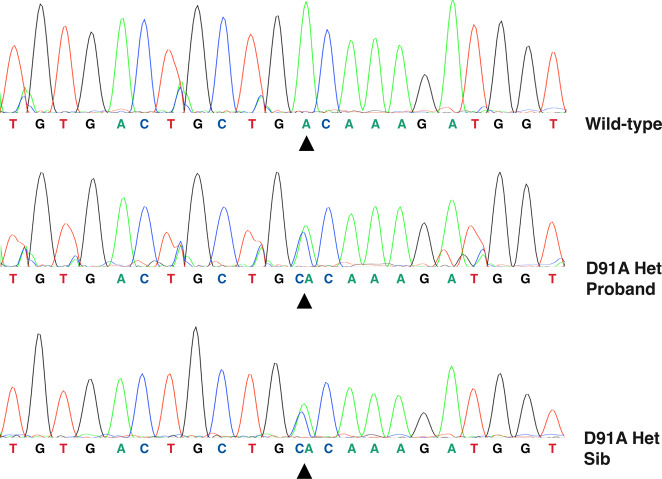
Partial *SOD1* gene sequence. Top panel shows wild-type DNA. Middle and bottom panels show a heterozygous cytosine to adenine change at position 272 (c.272A>C) in the ALS affected proband (II:1) and one unaffected sibling (II:10), respectively.

Whole-genome sequencing of the proband followed by Sanger confirmation revealed a heterozygous aspartic acid to alanine substitution at codon 91 (GAC>GCC) in the *SOD1* gene (Fig. 3). No damaging variants were found in genes associated with ALS including *C9orf72*, *TARDBP* or *FUS*. The same change was identified in three additional family members (age range, 74–76 years) (Figs. 2 and 3). On examination, none had neurological symptoms except for one brother (II:10) who reported difficulties in his handwriting (ALSFRS-R = 46). None of the healthy controls recruited from Gozo (11.1%) and mainland Malta (88.9%) were found to carry the *SOD1* D91A variant.

## Discussion

We describe the first *SOD1* variant in an ALS patient that has lived on the island of Gozo (Malta). Reported in many countries including in neighbouring Italy [6, 7, 16, 17], *SOD1* D91A is the most prevalent variant in Europe. Nonetheless, there seems to be a North-South gradient in the frequency of this allele across Europe (https://gnomad.broadinstitute.org/), with a high percentage reported in Finland and northern Sweden [10] and lower percentages characteristic of Mediterranean countries [6, 7]. ALS has been described in both *SOD1* D91A homozygotes and heterozygous carriers [4, 5], and reasons proposed, including the presence of genetic and/or environmental modifiers that decrease or increase susceptibility, remain debatable. Nevertheless, this conundrum underscores the need to be cautious in the genetic counselling of individuals carrying this variant.

It is noteworthy that sensory impairments described in our patient were also observed in heterozygous *SOD1* D91A ALS patients reported in Italy [16, 17] including one from Southern Italy [6]. This raises the question of whether this atypical presentation is characteristic of *SOD1* D91A ALS heterozygotes observed in both Italy and Malta, two adjacent countries that experienced similar genetic influences across the centuries. Similar to our case, most *SOD1* D91A heterozygotes described in Italy, including those in the south [6, 7, 16, 17], appear to be sALS cases. In contrast, compared to these patients whose onset appeared between 41 and 54 years, the Gozitan patient that we described had a late age of onset with ALS symptoms first appearing at the end of the seventh decade. At the present time, ALS has not developed in any of the three heterozygous siblings of our index case although a possible increased difficulty with handwriting in one brother highlights the need for continual monitoring by our clinic of these relatively asymptomatic *SOD1* D91A carriers. Nevertheless, should these remain without symptoms, genome analyses are warranted to identify protective genetic modifiers. In conclusion, our study underlies the relevance of *SOD1* variants to the population of Malta, particularly the potential role of D91A as an ALS susceptibility variant. This has implications for genotyping and future genotype-specific treatments.

## Data Availability

All data generated or analysed during this study are included in this published article.

## References

[1] van Es MA, Hardiman O, Chio A, Al-Chalabi A, Pasterkamp RJ, Veldink JH (2017). Amyotrophic lateral sclerosis. Lancet..

[2] Zou ZY, Zhou ZR, Che CH, Liu CY, He RL, Huang HP (2017). Genetic epidemiology of amyotrophic lateral sclerosis: a systematic review and meta-analysis. J Neurol Neurosurg Psychiatry.

[3] Rosen DR, Siddique T, Patterson D, Figlewicz DA, Sapp P, Hentati A (1993). Mutations in Cu/Zn superoxide dismutase gene are associated with familial amyotrophic lateral sclerosis. Nature..

[4] Andersen PM, Nilsson P, Ala-Hurula V, Keranen ML, Tarvainen I, Haltia T (1995). Amyotrophic lateral sclerosis associated with homozygosity for an Asp90Ala mutation in CuZn-superoxide dismutase. Nat Genet.

[5] Robberecht W, Aguirre T, Van den Bosch L, Tilkin P, Cassiman JJ, Matthijs G (1996). D90A heterozygosity in the SOD1 gene is associated with familial and apparently sporadic amyotrophic lateral sclerosis. Neurology..

[6] Battistini S, Giannini F, Greco G, Bibbo G, Ferrera L, Marini V (2005). SOD1 mutations in amyotrophic lateral sclerosis. Results from a multicenter Italian study. J Neurol.

[7] Ungaro C, Sprovieri T, Morello G, Perrone B, Spampinato AG, Simone IL (2021). Genetic investigation of amyotrophic lateral sclerosis patients in south Italy: a two-decade analysis. Neurobiol Aging.

[8] Al-Chalabi A, Andersen PM, Chioza B, Shaw C, Sham PC, Robberecht W (1998). Recessive amyotrophic lateral sclerosis families with the D90A SOD1 mutation share a common founder: evidence for a linked protective factor. Hum Mol Genet.

[9] Parton MJ, Broom W, Andersen PM, Al-Chalabi A, Nigel Leigh P, Powell JF (2002). D90A-SOD1 mediated amyotrophic lateral sclerosis: a single founder for all cases with evidence for a Cis-acting disease modifier in the recessive haplotype. Hum Mutat.

[10] Andersen PM, Forsgren L, Binzer M, Nilsson P, Ala-Hurula V, Keranen ML (1996). Autosomal recessive adult-onset amyotrophic lateral sclerosis associated with homozygosity for Asp90Ala CuZn-superoxide dismutase mutation. A clinical and genealogical study of 36 patients. Brain..

[11] Miller T, Cudkowicz M, Shaw PJ, Andersen PM, Atassi N, Bucelli RC (2020). Phase 1-2 trial of antisense oligonucleotide Tofersen for SOD1 ALS. N Engl J Med.

[12] Cassar C. A concise history of Malta. Valletta: Mireva Publications; 2000.

[13] Cauchi MN. A picture of Gozo: studies on ethnographic, educational and health aspects of life in Gozo. Malta: Gozo Press; 1998.

[14] Borg R, Farrugia Wismayer M, Bonavia K, Farrugia Wismayer A, Vella M, van Vugt J (2021). Genetic analysis of ALS cases in the isolated island population of Malta. Eur J Hum Genet.

[15] Farrugia Wismayer M, Borg R, Farrugia Wismayer A, Bonavia K, Vella M, Pace A, et al. Occupation and amyotrophic lateral sclerosis risk: a case-control study in the isolated island population of Malta. Amyotroph Lateral Scler Frontotemporal Degener. 2021:1–7. 10.1080/21678421.2021.1905847. 10.1080/21678421.2021.1905847 33821701

[16] Giannini F, Battistini S, Mancuso M, Greco G, Ricci C, Volpi N (2010). D90A-SOD1 mutation in ALS: the first report of heterozygous Italian patients and unusual findings. Amyotroph Lateral Scler.

[17] Origone P, Caponnetto C, Mascolo M, Mandich P (2009). Heterozygous D90A-SOD1 mutation in an Italian ALS patient with atypical presentation. Amyotroph Lateral Scler.

